# Dr Alain Cribier—The Man, the Myth, the Legend—January 25, 1945, to February 16, 2024

**DOI:** 10.1016/j.jscai.2024.101860

**Published:** 2024-03-05

**Authors:** Vasilis C. Babaliaros, Deborah C. Nercolini, Carla Agatiello, Raphael Amor

**Affiliations:** a Emory Structural Heart & Valve Center, Emory Healthcare, Atlanta, Georgia; b Instituto de Neurologia e Cardiologia de Curitiba, Curitiba, Brazil; c Hospital Italiano de Buenos Aires, Buenos Aires, Argentina; d Former employee of Percutaneous Valve Technologies and Edwards Lifesciences

Most of us have not had the privilege to meet our heroes. Whether fictional, figures from the past, or out of reach from our social circles, these larger-than-life characters are often not accessible to mere mortals. Alain Cribier was one of these figures whose impact on the lives of those around him, his students, friends, and patients, is the stuff of legend.

Man’s search for meaning is well documented in the manuscripts of scientific journals. In 2002, as a cardiology fellow, I was lost and struggling with the trajectory my career would follow. I spent hours reading journals, looking for something, and often struggling to stay awake. When I came across an article^1^ describing the first-in-human transcatheter aortic valve replacement (TAVR), I suddenly felt my purpose snap into focus. This was the beginning of my relationship with Alain Cribier.

**Figure 1:**
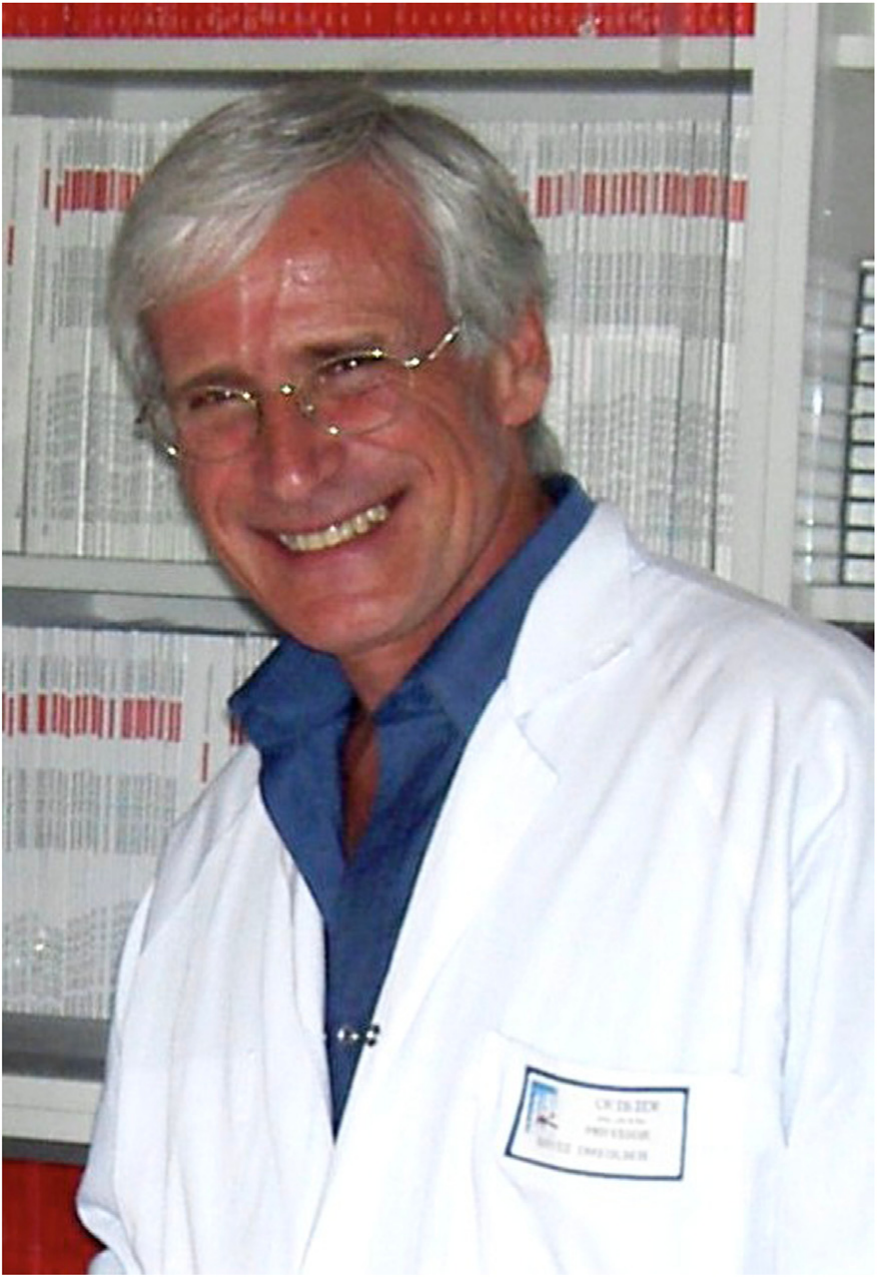
**Alain Cribier in 2004**.

Alain was born in Paris and trained as a cardiologist in France, but other than this, his story was not typical. He spent time in the United States studying under Drs Jeremy Swan and William Ganz but had also pursued spiritual enlightenment in India, an exercise that cured his hubris. He had a passion for medicine but a love of art and music that was equally as intense. His performance of Rachmaninoff concertos was astonishing to other doctors and even to musicians. The man I came to know was complicated—passionate, humble, dexterous, superhero, and human (Figure 1).

“We specialize in the paranormal,” he told me in one of our first encounters. I had just witnessed TAVR implant #16. I was coming out of a top tier academic training program for interventional cardiology in the US, and I could not comprehend exactly what I had seen. All I understood was Alain did not walk—he floated above the ground. He did not move catheters, he played piano in the cath lab. He could understand his present situation very quickly, but I was convinced he could also see the future.

**Figure 2:**
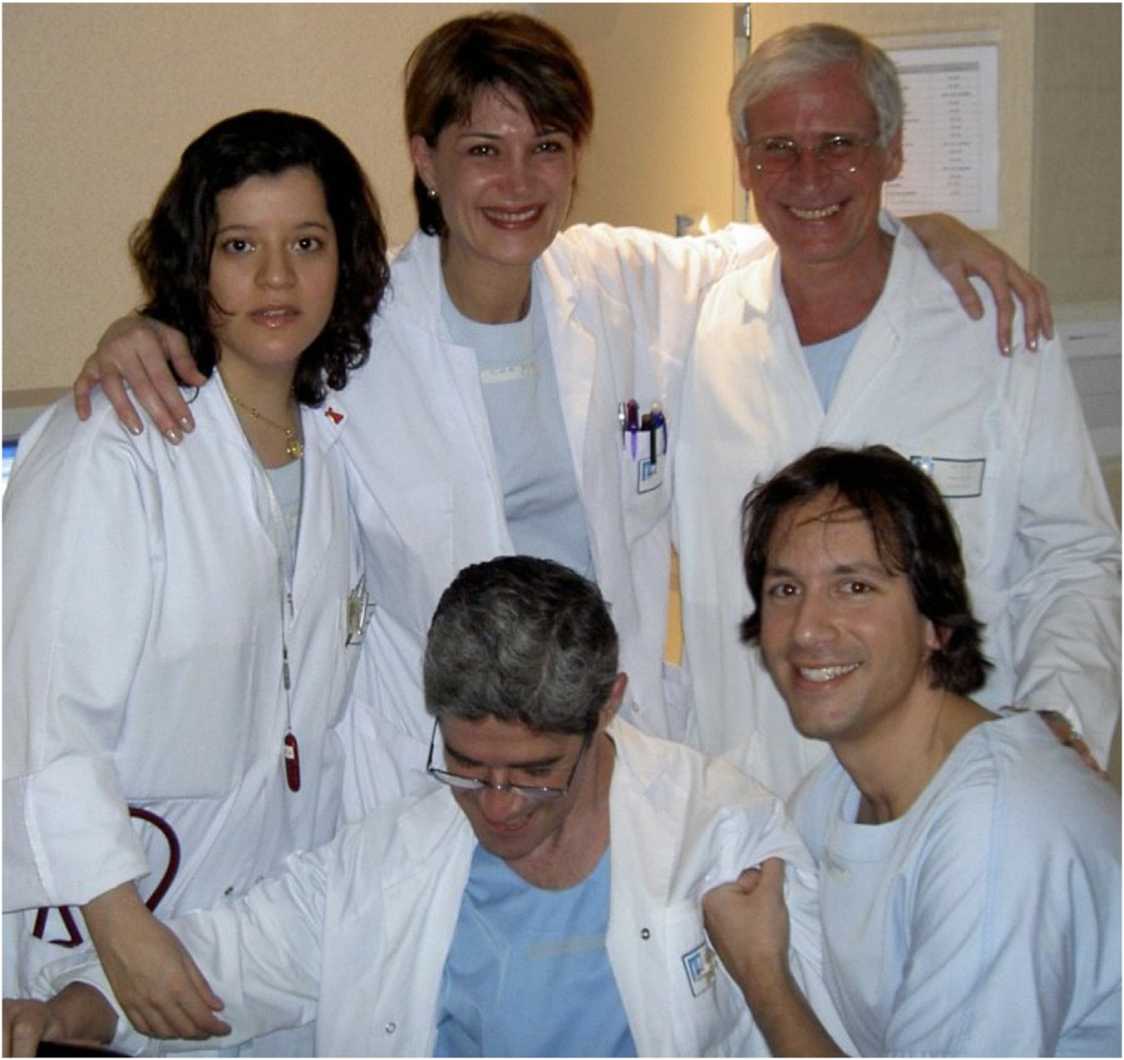
**An emotional ending to a successful transcatheter aortic valve replacement in 2004.** Rear: Carla Agatiello, Deborah C. Nercolini, and Alain Cribier. Front: Raphael Amor and Vasilis C. Babaliaros.

I would sign on to be his first North American fellow, joining 2 previous fellows who stayed on for the valve project, Deborah Nercolini and Carla Agatiello (Figure 2). Along with Raphael Amor, we would form the support system for the implanting team—Alain, Helene Eltchaninoff, and Christophe Tron. He later would be joined by Eric Durand and others. We worked days, nights, and weekends on manuscripts, presentations, correspondence, patient preparation, and research data because we believed we could speed up the evolution of TAVR. Alain never asked us to work harder; we did it because we loved him, and he instilled belief in our purpose.

You see, Alain was more than a boss to us. He was a mentor, friend, advocate, and first-class prankster. He was mischievous with a lightning-quick sense of humor in French and English, and wherever he went, he held court and the rest listened intently. The lessons were in philosophy, medicine, and art, followed by eruptions of laughter and horseplay. They occurred in the cath lab, in the doctor’s cafeteria (where the team ate lunch together daily at Alain’s insistence), and on the road. The traveling was intense that year. We took group trips to all the French meetings, and many of us were dispensed to regional meetings in Europe, Asia, and South America to cover the speaking invitations (often last-minute arrangements) with a complex strategy of planes, trains, and automobiles to get from hospital to destination. No one traveled more than Alain, who believed very firmly in medical education and communication. His schedule was very committed, and for that year he only slept well on planes. He would recount stories where passengers would try to converse with him before take-off, resulting in him throwing a blanket over his head midsentence so he could get some much-needed rest.

At the end of his trips, we always looked forward to his return to the Charles Nicolle Hospital. His stories and observations were insightful and humorous. He continually sifted through new ideas that would come to him as he traveled. He would stare patiently over his glasses at us as we tried to give him frantic updates and share our simple ideas back with him. He always had an infectious smile and was the forever optimist. We picked up his mannerisms, his cadence in speaking, and even tried to emulate his speed and excitement during procedures. When I returned to the US, I bought a yellow and black lead apron to channel his spirit when I worked. TAVR remained a sacred procedure, always under the watch of his sharp eyes. We tried to honor him by ensuring each procedure went well, and when it did not go well, we hoped to learn something to move us forward in the future. He instilled in many new trainees the need to challenge dogma as well as the need for innovation. We were driven to solve unmet needs for patients, and he was insistently optimistic we could be successful.

Social media is full of posts from people who have taken pictures with him over the years at numerous meetings. Alain continued to post on social media, present at meetings and performed TAVR occasionally at his teaching courses in Rouen even into his last weeks. He never gave up as a young man when he was fiercely opposed by colleagues for his early work in balloon aortic valvuloplasty and mitral commissurotomy. He continued to innovate as he got older and insisted on remaining relevant. He has been honored the world over, and despite the early nay-sayers, he never gave up on his dream to revolutionize treatment for valvular heart disease. Even his passionate battle with his health was classic Alain, always outsmarting and cheating death up until his final days, and then exiting like a bolt of lightning.

At a time when I was looking for meaning in my work, I serendipitously met my hero who showed me how to properly struggle for a greater good. And like the heroes in Greek mythology whose reward after death was immortality, Alain lives on in all of us, every time a TAVR valve is implanted or a new doctor dares to dream of a better way.
